# ICAM-1 Binding Rhinoviruses Enter HeLa Cells via Multiple Pathways and Travel to Distinct Intracellular Compartments for Uncoating

**DOI:** 10.3390/v9040068

**Published:** 2017-04-01

**Authors:** Haleh Ganjian, Christin Zietz, Diana Mechtcheriakova, Dieter Blaas, Renate Fuchs

**Affiliations:** 1 Department of Pathophysiology and Allergy Research, Medical University of Vienna, Währinger Gürtel 18-20, A-1090 Wien, Austria; halehganjian85@gmail.com (H.G.); a1368365@unet.univie.ac.at (C.Z.); diana.mechtcheriakova@meduniwien.ac.at (D.M.); 2 Max F. Perutz Laboratories, Department of Medical Biochemistry, Medical University of Vienna, Dr. Bohr Gasse 9/3, A-1030 Vienna, Austria; dieter.blaas@meduniwien.ac.at

**Keywords:** human rhinoviruses, ICAM-1, productive uncoating, endocytosis, clathrin, dynamin, macropinocytosis, actin, dynein

## Abstract

Of the more than 150 human rhinovirus (RV) serotypes, 89 utilize intercellular adhesion molecule-1 (ICAM-1) for cell entry. These belong either to species A or B. We recently demonstrated that RV-B14 and RV-A89, despite binding this same receptor, are routed into distinct endosomal compartments for release of their RNA into the cytosol. To gain insight into the underlying mechanism we now comparatively investigate the port of entry, temperature-dependence of uncoating, and intracellular routing of RV-B3, RV-B14, RV-A16, and RV-A89 in HeLa cells. The effect of various drugs blocking distinct stages on the individual pathways was determined via comparing the number of infected cells in a TissueFaxs instrument. We found that RV-B14 and RV-A89 enter via clathrin-, dynamin-, and cholesterol-dependent pathways, as well as by macropinocytosis. Drugs interfering with actin function similarly blocked entry of all four viruses, indicating their dependence on a dynamic actin network. However, uniquely, RV-A89 was able to produce progeny when internalized at 20 °C followed by neutralizing the endosomal pH and further incubation at 37 °C. Blocking dynein-dependent endosomal transport prevented uncoating of RV-A16 and RV-A89, but not of RV-B3 and RV-B14, indicative for routing of RV-A16 and RV-A89 into the endocytic recycling compartment for uncoating. Our results call for caution when developing drugs aimed at targeting entry or intracellular trafficking of all rhinovirus serotypes.

## 1. Introduction

Rhinoviruses (RVs) are the main cause of the common cold. Due to the large number of different serotypes (currently about 150 identified) infections are recurrent and, so far, no antiviral drug has reached the market. As members of the large family *Picornaviridae*, genus *Enteroviruses*, they feature a ~30 nm icosahedral capsid built from 60 copies each of four viral proteins that encases a single-stranded positive-sense RNA genome of about 7200 bases. Historically, rhinoviruses recognizing ICAM-1 (89 types) were called the major receptor group and those recognizing the low-density lipoprotein receptor (LDLR) family (12 types) were called the minor receptor group. The former are either species A or B, the latter are exclusively species A [1,2,3]; a more recently discovered species C [4], instead, uses cadherin-related family member 3 (CDHR3), a protein only marginally expressed in established tissue culture cells [5], and neither ICAM-1 nor LDLR. All RV-A and -B investigated so far are taken up by receptor-mediated endocytosis [6].

The minor receptor group species A prototype strain, RV-A2, enters HeLa cells preferentially via clathrin-mediated endocytosis [7] and uncoats in endosomal carrier vesicles (ECV)/late endosomes, a process strictly dependent on acidic pH [8,9]. Unlike LDLR, ICAM-1 facilitates uncoating in vitro, and in acidic compartments in vivo, to various degrees depending on the RV type [2,10,11,12]. At least for RV-B14, RNA penetration takes place from endosomal compartments (ECV or late endosomes) en route to lysosomes [13]. Recently, we found that RV-A89, despite also binding ICAM-1, is routed to the endocytic recycling compartment (ERC) for uncoating [14]. We, thus, wondered whether this was a unique property of this particular serotype and, if so, in what respect RV-A89 differed from other species A and B rhinoviruses. By using additional representatives of the A and B species, we now show that the profiles of chemical inhibitors blocking various stages of entry were virtually identical for RV-B14 and RV-A89. However, out of the four studied RVs, RV-A89 could also uniquely infect when internalized at 20 °C followed by neutralization of the endosomal pH and incubation at 37 °C. Blocking dynein-dependent transport to the minus end of microtubules, we demonstrate that RV-A16 and RV-A89, but not RV-B3 and RV-B14, are routed to the ERC.

## 2. Materials and Methods

### 2.1. Cell Culture, Virus Propagation, and Reagents

HeLa-H1 (in the following termed ‘HeLa’) were from ATCC (Manassas, VA, USA) and grown in minimum essential medium (MEM) supplemented with 10% heat-inactivated foetal calf serum (FCS), 2 mM l-glutamine, 100 units/mL penicillin G sodium salt, and 100 µg/mL streptomycin sulphate (all from Gibco Invitrogen Corp., Paisley, UK). RV-B3, RV-B14, RV-A16, and RV-A89 were originally obtained from ATCC. The virus titres were determined as TCID_50_ (50% tissue culture infectious dose; [15]). For virus internalization, serum-free MEM+ (MEM containing 30 mM MgCl_2_ and 1% l-glutamine) was used; viruses were grown in cells maintained in infection medium (MEM containing 2% FCS, 1% l-glutamine, 30 mM MgCl_2_). All chemicals were from Sigma (St. Louis, MO, USA), unless specified otherwise. Bafilomycin A1 (Alexis Corp., Lausen, Switzerland) was dissolved in dimethylsulfoxid (DMSO) at 20 mM and stored at −20 °C. Except for chlorpromazine, which was at 100 mM in phosphate buffered saline (PBS), all other drugs were dissolved in DMSO at the following concentrations: dynasore, 80 mM; filipin 5 mg/mL (7.6 mM); cytochalasin D, 10 mg/mL (20 mM); blebbistatin, 20 mM; jasplakinolide, 1 mM; ciliobrevin, 30 mM. Final concentrations in the assays were for dynasore, 80 µM; chlorpromazine, 100 µM; filipin, 5 µg/mL; cytochalasin D, 10 µg/mL (20 µM); blebbistatin, 20 µM; jasplakinolide, 1 µM; ciliobrevin, 30 µM; bafilomycin, 200 nM.

### 2.2. Measuring the Effect of Inhibitors on RV Productive Uncoating

HeLa cells grown on coverslips were pre-incubated in serum-free MEM+ with the respective solvent (control), ±inhibitor or ±25 mM NH_4_Cl, respectively. RV-B3, RV-B14, RV-A16, or RV-A89 (all at 100 TCID_50_/cell) were then internalized at 37 °C for 60 min, likewise in the presence or absence of the same drugs. The medium containing drug and non-attached virus was removed, cells were washed with cold PBS containing 1 mM CaCl_2_ and 1 mM MgCl_2_ (PBS+) plus 25 mM NH_4_Cl for 15 min on ice, transferred to warm infection medium containing 25 mM NH_4_Cl, and further incubated for 7 h at 37 °C. These conditions allow for the replication of the viruses that had uncoated prior to the addition of NH_4_Cl. Cells were fixed with 4% paraformaldehyde (PFA) in PBS, quenched with 50 mM NH_4_Cl in PBS, permeabilized with 0.2% Triton X-100, and blocked with 10% goat serum in PBS. The double-stranded RNA (dsRNA) produced during virus replication was detected with monoclonal antibody mAbJ2 at 1:3000 (0.66 µg/mL; English and Scientific Consulting, Bt. Szirák, Hungary) in 10% goat serum for 1 h at room temperature [16]. The mouse antibody was then revealed with Alexa Fluor® 488 goat anti-mouse IgG (Life Technologies, Vienna, Austria) at 1 μg/mL. Nuclei were labelled with 4′,6-diamidine-2′-phenylindole DAPI (Sigma Aldrich, St. Louis, MO, USA; 1 µg/mL) for 10 min. Coverslips were mounted with 4 μL Fluoromount-G (SouthernBiotech, Birmingham, AL, USA. Images were recorded on a TissueFaxs automated microscope (TissueGnostics, Vienna, Austria) and the numbers of infected cells relative to the control incubation without drugs (arbitrarily set to 100%) were determined using the TissueQuest 3 software [17].

### 2.3. Internalization of RVs in the Presence of Filipin 

HeLa cells on coverslips were pre-incubated with serum-free MEM+ containing 5 µg/mL filipin. The positive control contained only DMSO (1%). RV-B14 and RV-A89 at 100 TCID_50_/cell were internalized at 37 °C for 60 min in the absence or presence of the same drug. The medium containing drugs and non-attached viruses was removed, cells were washed with cold PBS+, fixed for 20 min in 4% PFA, and quenched for 10 min with 50 mM NH_4_Cl. Non-specific binding sites were blocked by incubation for 1 h in 10% goat serum. ICAM-1 receptors at the plasma membrane were stained with mouse anti-ICAM-1 antibody R6.5 (eBioscience, Vienna, Austria; 1 µg/mL) followed by Alexa Fluor® 488 goat anti-mouse IgG. Cells were then permeabilized for 10 min with 0.2% Triton X-100 in PBS and stained for RV-B14 and RV-A89, as described, above using Alexa Fluor® 568-coupled secondary antibodies. Nuclei were revealed by using DAPI (1:1000). Confocal images were acquired with a ZEISS LSM700 microscope using ZEN software (Jena, Germany).

### 2.4. Measuring Uncoating at 20 °C

Cells grown on coverslips were pre-incubated in serum-free MEM+ at 37 °C for 30 min. RV-B3, RV-B14, RV-A16, and RV-A89 (all at 100 TCID_50_/cell) were internalized at 37 °C for 60 min or at 20 °C for 120 min, to compensate for the lower uptake efficiency. They were then washed with cold PBS+ containing 25 mM NH_4_Cl, transferred into infection medium plus 25 mM NH_4_Cl, and further incubated for 7 h at 37 °C. Replication was then detected in individual cells via the presence of dsRNA as above. The percentage of cells becoming infected following virus entry at 37 °C for 60 min was arbitrarily set to 100% for each serotype.

### 2.5. Determination of the Generation of Modified Viral Particles upon Internalization at 20 °C

HeLa cells grown in 24-well plates were pre-incubated for 30 min with MEM+ without or with 200 nM bafilomycin. Subsequently, RV-B3, RV-B14, RV-A16, or RV-A89 (all at 100 TCID50) were internalized in MEM+ with or without 200 nM bafilomycin for 2 h at 20 °C. Cells were then cooled and the supernatant media were removed and replaced with fresh, ice-cold MEM+. Cells were subjected to three freeze/thaw cycles and the infectious virus in each well was determined as TCID50 [15].

### 2.6. Uncoating from the Plasma Membrane

Isotonic buffers were prepared by adjusting a mixture of 30 mM sodium acetate, 10 mM sodium phosphate (pH 5.0), 110 mM NaCl, and 5 mM KCl with 0.1 M NaOH to pH values of 7.4 and 5.3. Cells were grown on coverslips and pre-incubated with or without 200 nM bafilomycin in serum-free MEM+ at 37 °C for 30 min. RV-B14 and RV-A89 (at 100 TCID_50_/cell) were allowed to bind to the cells at 4 °C for 60 min. The cells were washed, and incubated in the above isotonic buffers of pH 7.4 (with 200 nM bafilomycin and without (control)) or of pH 5.3 with 200 nM bafilomycin [8] for 1 h at 4 °C. They were then washed with cold PBS+ (±200 nM bafilomycin), transferred to warm infection medium (±200 nM bafilomycin), and further incubated for 16 h at 37 °C to allow for replication. Cells were fixed and processed for indirect immuno-fluorescence microscopy as above. The following antibody combinations and dilutions were used: (i) for RV-A2, mouse antibody 8F5 [18] (1.45 μg/mL) and Alexa Fluor® 488 goat anti-mouse IgG (1 μg/mL); (ii) for RV-B14, rat antiserum [14] (1:2000) and Alexa Fluor® 488 goat anti-rat IgG (1 μg/mL); and (iii) for RV-A89, rabbit antibody P5 (1:1000) and Alexa Fluor® 568 goat anti-rabbit IgG (1 μg/mL). Since no specific antiserum was available, RV-B16 was not included. Nuclei were stained with DAPI (1:1000) for 10 min. Coverslips were mounted with 4 μL Fluoromount-G and images were recorded as above.

### 2.7. Effect of RV Entry on the Actin Cytoskeleton

Cells grown on coverslips were pre-incubated with MEM+ for 30 min at 37 °C. The respective virus (at 100 TCID_50_/cell in MEM+) was then internalized for 5 min, 10 min, 15 min, or 30 min at 37 °C. Cells were fixed, permeabilized, and processed for indirect immunofluorescence microscopy as above. Actin filaments were stained with Alexa Fluor® 568 phalloidin (Life Technologies, Vienna, Austria) according to the manufacturer’s protocol. Nuclei were stained with DAPI (1:1000). Confocal images were acquired with a ZEISS LSM700 microscope using ZEN software.

### 2.8. Effect of Overexpression of Dominant-Negative Mutants of the C-Terminal Domain of AP-180 (AP180-C) and the SH3 Domain of Amphiphysin (amp-SH3) on Viral Uptake and Infectivity

Cells were grown on coverslips in 24-well plates until 70% confluent and separately transfected with the dominant-negative mutants AP180-C and Amp-SH3, which are both myc-tagged at the C-terminus [7,19,20]; OptiMEM (50 µL) was mixed with 250 ng plasmid DNA and 0.25 µL PLUS reagent. Lipofectamine LTX (1.25 µL; Thermo Fisher Scientific, Vienna, Austria) was mixed with 50 µL OptiMEM, and the solutions were combined and incubated for 30 min at room temperature. After addition of 400 µL serum-free MEM, the mixture was added to the cells. On the following day, the transfected cells were pre-incubated with MEM+ and virus was allowed to internalize for 60 min at 37 °C at 100 TCID_50_/cell in 200 µL MEM+. Cells were cooled and unbound viruses were removed. Alternatively, cells were incubated in infection medium (containing 25 mM NH_4_Cl) for 7.5 h at 37 °C. The cells were processed for indirect immunofluorescence microscopy using the following antibodies: (i) for RV-A89, rabbit antibody P5 (1:1000), and Alexa Fluor® 568 goat anti-rabbit IgG (1 µg/mL); and (ii) for RV-B14, rat anti-RV-B14 antiserum [14], and Alexa Fluor® 568 goat anti-rat IgG (1 µg/mL). The dominant-negative mutants were detected with mouse anti-myc antibody clone 4A6 (1:1000) and Alexa Fluor® 488 goat anti-mouse IgG (1 µg/mL). Nuclei were stained by either using DAPI (1:1000) or DRAQ 5 (at 10 µM; Thermo Scientific, Rockford, IL, USA) and coverslips were mounted with 4 µL Fluoromount on glass slides. To investigate virus internalization, confocal images were acquired with a Perkin Elmer Ultraview ERS rapid confocal imager using Volocity 6.1 software (Mannheim, Germany). Z stacks were spaced by 0.4 µm. Images were recorded on a TissueFaxs automated microscope (TissueGnostics) and the numbers of infected cells relative to the cells not expressing AP180-C or amp-SH were arbitrarily set to 100%.

## 3. Results

### 3.1. Internalization of RV-B14 and RV-A89 Occurs via Multiple Pathways

**Figure 1:**
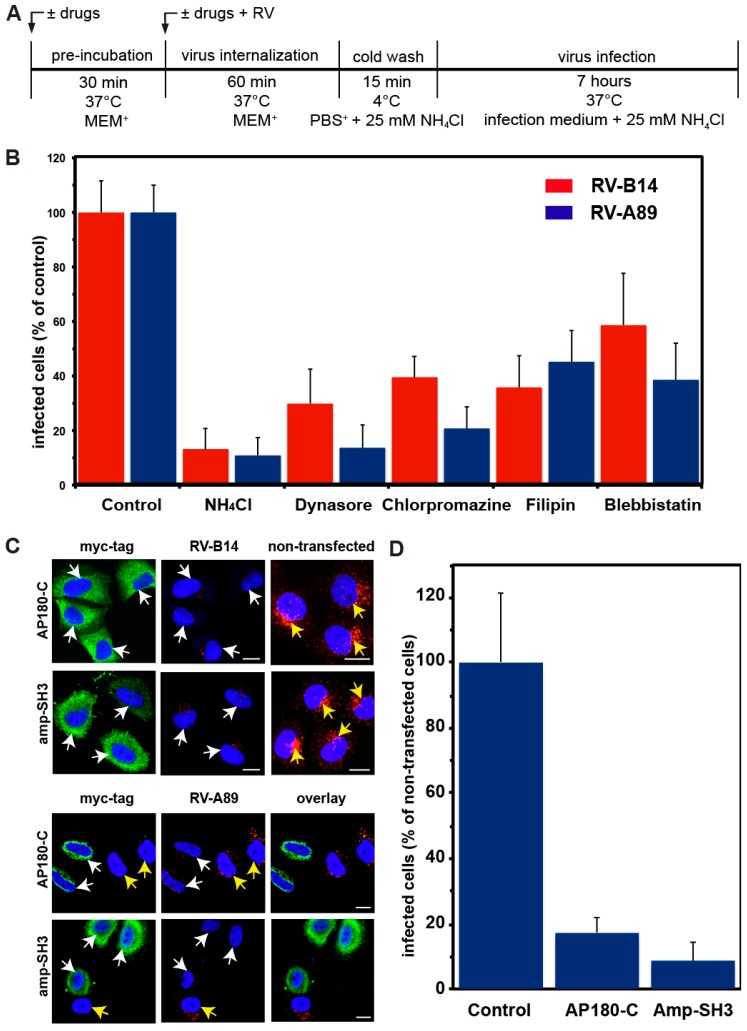
RV-B14 and RV-A89 enter HeLa cells via multiple pathways. The experimental setup is depicted in (**A**). After pre-incubating cells ± drugs (NH_4_Cl, dynasore, chlorpromazine, filipin, or blebbistatin), the respective virus at 100 TCID_50_/cell (50% tissue culture infectious dose/cell) was internalized for 60 min ± drugs. Non-internalized viruses were washed off with ice-cold phosphate buffered saline containing 1 mM CaCl_2_ and 1 mM MgCl_2_ (PBS+) plus NH_4_Cl to raise the endosomal pH and thereby stop further virus uncoating. Cells were transferred into infection medium containing 25 mM NH_4_Cl and dsRNA stemming from replicating viruses was detected with mAbJ2, followed by Alexa-488 goat-anti mouse IgG. Nuclei were stained with 4′,6-diamidino-2-phenylindol DAPI. The number of cells revealing mAbJ2 staining was determined in a TissueFaxs and related to the total number of cells. Infected cells in the absence of any drug were set to 100%. In each sample about 500,000 cells were analysed. (**B**) Data shown are the mean (± standard deviation (SD)) of five separate experiments each carried out with five parallels. The presence of NH_4_Cl throughout the incubation served as negative control. In all experiments shown (also in Figure 2, Figure 3, Figure 4 and Figure 5), NH_4_Cl blocked viral uncoating by 80–90%. Based on the inhibitory effect of dynasore, chlorpromazine, filipin, and blebbistatin, RV-B14 and RV-A89 enter HeLa cells by dynamin, clathrin, and cholesterol-dependent pathways, as well as by macropinocytosis. (**C**) HeLa cells were transfected with plasmids encoding myc-tagged dominant-negative mutants of AP-180 (AP180-C) and amphiphysin (amp-SH3), as indicated. After 24 h, RV-B14 or RV-A89 (100 TCID_50_/cell) was internalized for 60 min at 37 °C, cells were cooled, viruses in the supernatant medium were removed by washing, and myc-tagged proteins were stained with anti-myc antibodies (green) and viral proteins with rat anti-RV-14 antibody or antiserum P5, followed by the respective secondary antibodies (red). Bar, 10 µm. Cells expressing either AP180-C or amp-SH3 (white arrows) revealed reduced internalization of RVs as compared to non-transfected control cells (yellow arrows). (**D**) HeLa cells were transfected with plasmids encoding myc-tagged dominant-negative mutants of AP-180 (AP180-C) and amphiphysin (amp-SH3) as indicated. After 24 h, RV-A89 (100 TCID_50_/cell) was internalized for 60 min at 37 °C and the cells were further incubated for 7.5 h in infection medium containing 25 mM NH_4_Cl. Infected cells were identified with antibody P5 in a TissueFaxs and related to cells either expressing AP180-C or amp-SH3 (mean ± SD from five parallel samples), or neither. In agreement with RV-A89 internalization (**C**), productive uncoating of the viruses was reduced to 20% and 10% in cells overexpressing AP180-C and amp-SH3, respectively.

Since RV-B14 and RV-A89 differed in their kinetics of internalization and RNA release [14], we investigated whether this is due to them using different entry routes. To this end, we applied drugs that interfere with dynamin (dynasore) [21], clathrin (chlorpromazine) [22], caveolae (filipin) [23], or with macropinocytosis (blebbistatin) [24], and determined their effect on productive entry of these viruses, which we define as resulting in RNA uncoating and virus replication. Effects of the drugs on viral replication were excluded as follows: HeLa cells were pre-incubated without and with the respective inhibitor. The viruses were internalized for 1 h at 37 °C ± inhibitor, cells were cooled, and unbound viruses were washed away with ice-cold PBS+ containing 25 mM NH_4_Cl. The cells were transferred into infection medium containing 25 mM NH_4_Cl to prevent further uncoating. To allow for replication of the viral RNA released in the previous step, the cells were then maintained for 7 h at 37 °C, fixed, and processed for detection of dsRNA (stemming from replicating viruses) using mAbJ2 [16] (for experimental setup see Figure 1A). Infected cells were quantitated in a TissueFaxs [14] and related to the total number of cells identified by nuclear staining with DAPI. Control incubations with NH_4_Cl, which was maintained throughout the experiment, were always included. As shown in Figure 1B, all drugs inhibited entry of either virus, albeit to different degrees. Dynasore was the most potent inhibitor. Together with the inhibitory effect of chlorpromazine, this suggests that uptake is clathrin- and dynamin-dependent. This is in agreement with the results of DeTulleo and colleagues [25] who demonstrated that RV-B14 infection was blocked upon overexpression of non-functional dynamin K44A in HeLa cells.

To further investigate clathrin-mediated virus internalization, we overexpressed dominant-negative versions of AP180 (AP180-C) and amphiphysin (amp-SH3) in HeLa cells. The C-terminal domain of AP-180 interacts with clathrin heavy chains; overexpression of AP180-C blocks clathrin recruitment to the plasma membrane and, consequently, clathrin-mediated endocytosis [20]. Amphiphysin interacts with AP2 at its N-terminus and with the proline-rich domain of dynamin at its C-terminal SH3 domain [19]. Upon overexpression of amp-SH3, dynamin cannot be recruited to clathrin-coated pits. Following transfection of HeLa cells with the plasmids encoding myc-tagged AP180-C and myc-tagged amp-SH3, respectively, RV-B14 and RV-A89 were incubated with the cells for 1 h at 37 °C, unbound viruses were removed, and internalized viruses were analysed by immunofluorescence microscopy. As shown in Figure 1C, cells overexpressing AP180-C or amp-SH3 were strongly reduced in intracellular staining of RV-B14 or RV-A89. Furthermore, productive uncoating of RV-A89, as analysed by de novo synthesized viral proteins, was reduced to 20% and 10%, respectively (Figure 1D). This extends the finding of DeTulleo et al. in that RV-A89 entry is also dynamin and clathrin-dependent [25].

**Figure 2:**
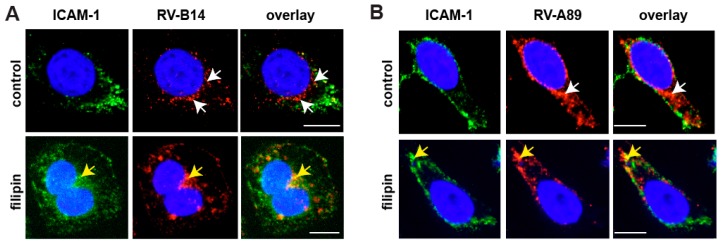
Immunofluorescence localization of RV-B14 (**A**) and RV-A89 (**B**) in the absence (control) and in the presence of filipin. Viruses were internalized into HeLa cells as indicated, cells were cooled, fixed, and intercellular adhesion molecule-1 (ICAM-1) at the plasma membrane was stained with monoclonal antibody (mAb) R6.5 and Alexa-488-labelled secondary antibody. The cells were then permeabilized and RV-B14 and RV-A89 proteins were detected with the respective antibodies (see Materials and Methods). Confocal images are shown. Bar, 10 µm. In the absence of filipin, viral proteins are detected in endosomes in the perinuclear area (white arrows), whereas in the presence of the drug they co-localized with ICAM-1 at the plasma membrane (yellow arrows).

Filipin binds cholesterol from cellular membranes and, thus, disrupts lipid rafts and caveolae [23]. Inhibition of productive uncoating of both viruses by filipin suggests lipid rafts/caveolae as additional ports of entry and/or the requirements for cholesterol for RNA penetration into the cytosol. As shown by immunofluorescence microscopy, filipin prevented RV-B14 and RV-A89 internalization (Figure 2). Finally, virus uptake via macropinocytosis was investigated using amiloride and ethyl isopropyl-amiloride (EIPA) that block the activity of the Na+/H+ exchanger required for macropinosome formation [24]. However, both drugs elevated the endosomal pH in HeLa cells (data not shown), which does not allow for a valid conclusion. Thus, we used blebbistatin, an inhibitor of non-muscle myosin II [24], which is involved in membrane ruffling. As shown in Figure 1B, the drug reduced uncoating of both viruses by 40–60%, similarly suggesting a role of macropinocytosis in RV internalization. In conclusion, RV-B14 and RV-A89 can use various entry pathways to infect HeLa cells. For RV-B14, this is also in agreement with the virus being seen in uncoated and coated vesicles when viewed in an electron microscope [26,27].

### 3.2. Internalization of the Major Receptor Group RV-B3, RV-B14, RV-A16, and RV-A89 Is Dependent on Actin

**Figure 3:**
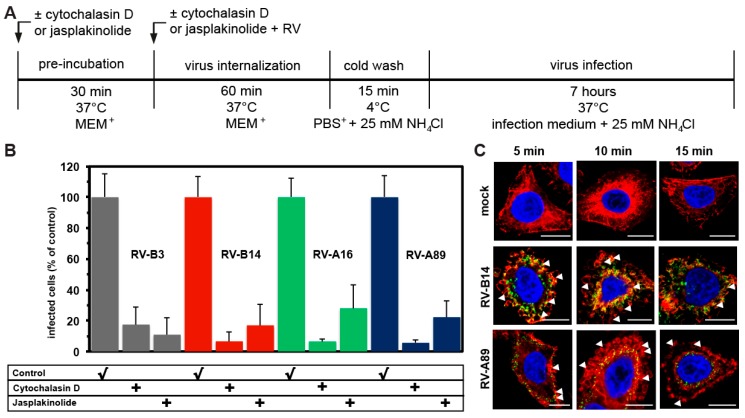
Entry of RV-B3, RV-B14, RV-A16, and RV-A89 requires a dynamic actin cytoskeleton. (**A**) Experimental setup. (**B**) Determination of RV dsRNA synthesis in the absence (control) or presence of cytochalasin D or jasplakinolide carried out as in Figure 1B. Data are the mean ± SD from five experiments, each carried out in quintuplicate. Productive uncoating of all viruses was inhibited to the level of the negative control (NH_4_Cl present throughout, compare to Figure 1A). (**C**) Virus-induced alteration of the actin cytoskeleton. RV-B14 and RV-A89 were internalized in minimum essential medium containing 30 mM MgCl_2_ and 1% L-glutamine (MEM+) for 5, 10, and 15 min. Cells were fixed, permeabilized, and stained for viral proteins as in Figure 2 (green). Actin filaments were stained with Alexa-568-phalloidin (red) and nuclei with DAPI (blue). Confocal images are shown. Bar, 10 µm. Virus internalization led to membrane ruffling (arrowheads), which was most pronounced at 10 min, suggesting that virus uptake occurs via macropinocytosis.

Previously, entry of RV-B14 and RV-A89 into early endosomes in HeLa cells was shown to require an intact actin network [28]. In contrast, clathrin-mediated internalization of the minor group virus RV-A2 was unaffected by the actin-depolymerizing drug cytochalasin D [26,28]. We, thus, analysed whether productive entry of the major receptor group RV-B3 and RV-A16 was also actin-dependent; on the one hand, actin filaments were depolymerized with cytochalasin D and, on the other hand, stabilized with jasplakinolide [29]. Both drugs strongly reduced productive entry of all viruses investigated to the level of the negative controls (Figure 3). Macropinocytosis is entirely actin-dependent. However, inhibition of virus entry was much more inhibited by drugs that interfere with actin cytoskeleton dynamics than by blocking macropinocytosis with blebbistatin. This may be due to virus-induced ICAM-1 association with the actin linker protein ezrin leading to signalling events required for RV entry and the release of inflammatory cytokines, as shown in primary human airway epithelial cells [30,31,32]. Nevertheless, our results can also be interpreted as evidence for major group virus uptake via macropinocytosis. Viruses known to enter cells by macropinocytosis induce membrane protrusions of various forms that can be seen by scanning electron microscopy, in differential interference contrast (DIC) microscopy and, upon staining of actin filaments with the fluorescent phalloidin, by fluorescence microscopy [24,33]. For analysis by the latter method, we internalized RV-B14 and RV-A89 for 5 min, 10 min, and 15 min. Cells were then fixed and actin was stained with Alexa-568 phalloidin. As shown in Figure 3C, RV-B14 and RV-A89 induced transient membrane blebbing, which was clearly visible at 5 min and 10 min, but not after 15 min. This indicates, concordant with the inhibitory effect of blebbistatin (Figure 1B) and cytochalasin D (Figure 3B), that macropinocytosis also plays a role in entry of at least these two virus types.

### 3.3. RV-A89, but None of the Three Other Major Group Viruses Studied Uncoats at 20 °C

**Figure 4:**
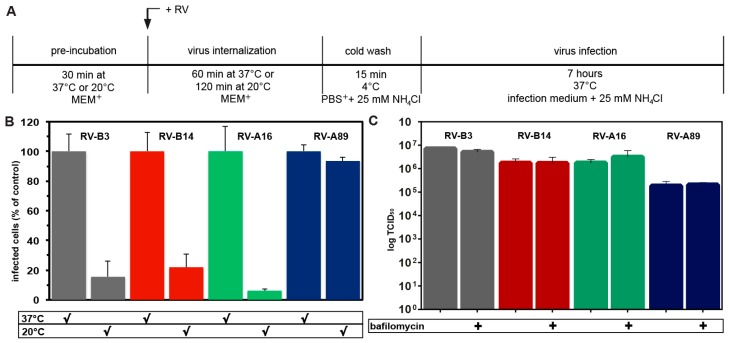
Only RV-A89 is able to replicate on challenge of HeLa cells at 20 °C when followed by neutralization of the endosomal pH and transfer to 37 °C. (**A**) Experimental setup. (**B**) The respective virus was internalized at 100 TCID50/cell either for 60 min at 37 °C (control) or for 120 min at 20 °C. Non-internalized virus was removed and cells were transferred into infection medium containing NH4Cl and incubated for 7 h at 37 °C to allow for its replication. Cells producing viral dsRNA were identified with mAbJ2 and quantified in a TissueFaxs, as in Figure 1B. Cells infected upon challenge at 20 °C as compared to 37 °C are given as percent. Data (mean ± SD) are from three individual experiments, each carried out in quintuplicate. Note that only RV-A89 was able to infect under this particular condition. (**C**) Virus internalized at 20 °C remains infectious. Virus was internalized into HeLa cells grown in 24 well plates for 120 min at 20 °C in the absence and presence of 200 nM bafilomycin. Cells were than cooled, the supernatants were removed, fresh, cold MEM+ was added, cells were subjected to repeated freezing and thawing, and infectious virus was determined. Data are the mean ± SD of five parallel samples. Note that the viral titre was virtually unchanged at 120 min internalization at 20 °C regardless of the presence of bafilomycin.

The minor group virus RV-A2 converts into subviral particles recognized by the specific mAb 2G2 even when internalized at 20 °C [8,34]. At this temperature, fusion between late endosomes and lysosomes does not occur, but the virus similarly arrives in early and late endosomes, albeit more slowly. On the other hand, transferrin transport to the ERC and its recycling to the plasma membrane are only marginally disturbed at 20 °C [35]. The above major group viruses were, thus, internalized for 1 h at 37 °C (control) or for 2 h at 20 °C. To allow for subsequent viral replication (i.e., as a consequence of productive uncoating), NH_4_Cl was added to prevent uncoating of internalized, but still native, viruses, and the incubation temperature was raised to 37 °C (for experimental setup see Figure 4A). As a negative control, NH_4_Cl was kept present throughout the experiment. Cells synthesizing dsRNA were determined by immunofluorescence microscopy and quantitated in a TissueFaxs as above (Figure 4B). RV-A89, but none of the other serotypes, produced appreciable progeny virus under these particular conditions. For RV-B3, RV-B14, and RV-A16 (RV-A89 was not included in the study) this finding agrees with the temperature-dependence of in vitro uncoating [10]. However, when incubated with acetate buffer of various acidic pH values at 20 °C for 1 h, RV-A89 proved no more acid labile than the other viral serotypes (data not shown). This latter result also agrees with the fact that the titre of infectious virus recovered from the cells at 2 h of internalization at 20 °C was virtually the same regardless of presence or absence of bafilomycin (Figure 4C). Therefore, RV-A89 appears to be unique in undergoing some kind of ‘priming’ for RNA release at 20 °C. Since all virus types internalized at 20 °C remain infective, transformation of RV-A89 into empty capsids must take place upon warming to 37 °C, even in the presence of bafilomycin or NH_4_Cl. In this respect, RV-A89 is clearly distinct from the other three serotypes.

### 3.4. RV-B14 and RV-A89 Cannot Uncoat at the Plasma Membrane

**Figure 5:**
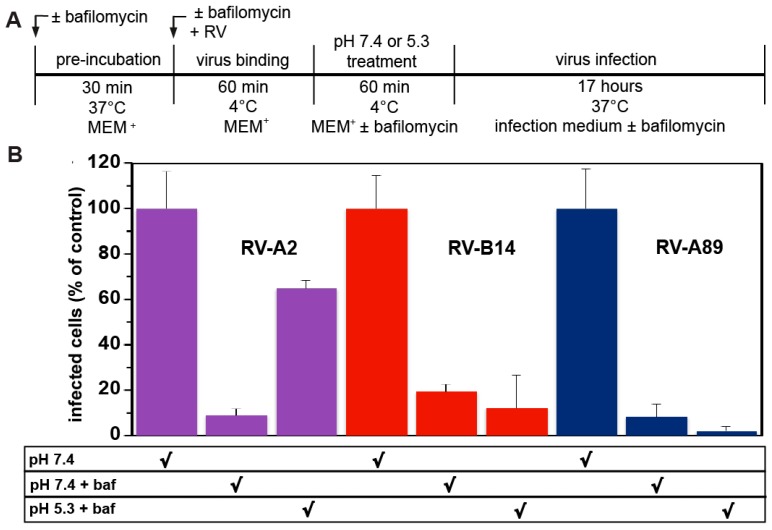
RV-B14 and RVA89 cannot uncoat at the plasma membrane upon low pH treatment. (**A**) Experimental setup. (**B**) HeLa cells were pre-incubated with MEM+ ± 200 nM bafilomycin, to neutralize the endosomal pH. Cells were than cooled and viruses at 100 TCID_50_/cell were bound for 60 min at 4 °C in the absence or presence of 200 nM bafilomycin. Unbound viruses were removed and cells were treated with isotonic buffer at pH 7.4 ± bafilomycin, and at pH 5.3 + bafilomycin, respectively. Cells were then transferred into infection medium + 200 nM bafilomycin for 17 h to allow for viral replication. Viral proteins were detected with mAb 8F5 (RV-A2), rat antiserum (RV-B14), and rabbit antiserum P5 (RV-A89), and the respective Alexa-488-labelled secondary antibodies. Infected cells were quantitated in a TissueFaxs. Cells infected via the endosomal route (pH 7.4 treatment in the absence of bafilomycin) were set to 100%. Values depicted are the mean ± SD from three independent experiments, each carried out in quintuplicate. RV-A2, but neither RV-B14 nor RV-A89, can productively uncoat via exposure to low pH at the plasma membrane.

RV-A2 converts into subviral particles when exposed to acidic pH even at 4 °C; when bound to the plasma membrane and treated with buffer of pH 5.3, we observed replication on warming to 34 °C in the presence of bafilomycin [36]. We, thus, asked whether the major group viruses RV-B14 and RV-A89 might also uncoat at the plasma membrane. As a control, RV-A2 was included in this experiment. The respective virus was bound to HeLa cells for 1 h at 4 °C in MEM+, pH 7.4 (Figure 5A). Unbound virus was removed and cells were treated for 1 h at 4 °C with isotonic buffer of pH 7.4 (±bafilomycin) or of pH 5.3 + bafilomycin. Cells were transferred into infection medium ± bafilomycin, incubated for 17 h, and those synthesizing viral proteins were quantified. HeLa cells infected via the endosomal route (pH 7.4, bafilomycin) were set to 100%. In agreement with earlier results, RV-A2 could release its RNA into the cytosol from the plasma membrane after treatment with acidic buffer. In contrast, neither RV-B14 nor RV-A89 was able to productively release its RNA at the plasma membrane into the cytosol (Figure 5B).

### 3.5. Uncoating of RV-B3 and RV-B14 Depends on Functional Dynein

**Figure 6:**
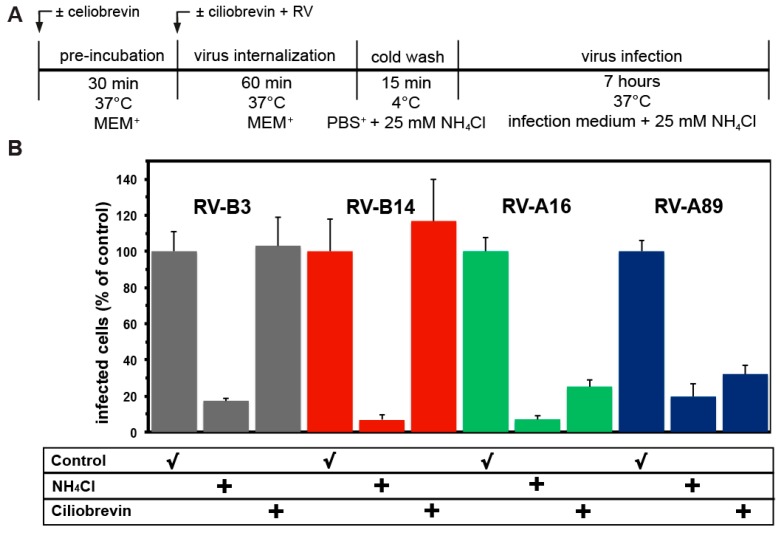
Ciliobrevin that blocks endosomal transport along microtubules inhibits uncoating of RV-A16 and RV-A89, but not of RV-B3 and RV-B14. (**A**) Experimental setup. (**B**) Viruses were internalized in the absence and presence of ciliobrevin for 60 min at 37 °C and dsRNA was determined with mAbJ2 as in Figure 1B. Data shown are the mean ± SD from five independent experiments, each carried out in quintuplicate. Ciliobrevin prevented productive uncoating of RV-A16 and RV-A89, indicative for routing of these serotypes to the endocytic recycling compartment (ERC). In contrast, productive uncoating RV-B3 and RV-B14 were unaffected by the drug.

As outlined in the introduction, RV-A89, but not RV-B14, uncoating depends on transport into the ERC [14]. This was shown by disrupting microtubules with nocodazole, by blocking dynein-mediated vesicular transport to the minus end of microtubules with ciliobrevin, and by a strong inhibition of infection by overexpression of dominant negative rab11 (see Figure 1 in [14]). Here we further extended these studies to RV-B3 and RV-A16, including RV-B14 and RV-A89 as controls. The experimental setup was identical to that shown in Figure 1A. As expected, RV-A89 uncoating was strongly inhibited by ciliobrevin (Figure 6B). A similar inhibitory effect was observed for RV-A16. However, RV-B3 uncoating was not affected by the drug. These data suggest that two major group A viruses with similarly high affinity for ICAM-1 [37] are routed to the ERC, whereas two major group B viruses with similarly low affinity for ICAM-1 release their RNA on the way through the lysosomal pathway.

## 4. Discussion

**Figure 7:**
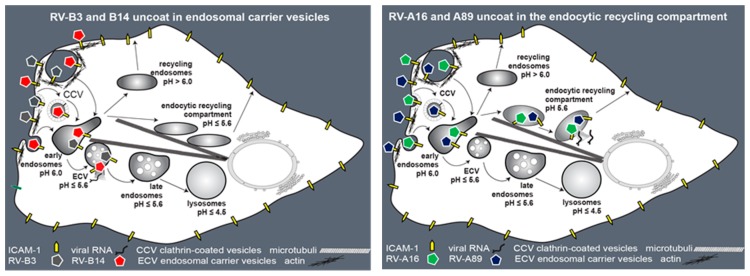
Schematic summary of our results. Endocytosis of RV-B3, RV-B14, RV-A16, and RV-A89 depends on a dynamic actin cytoskeleton. RV-B14 and RV-A89 enter HeLa cells via clathrin and dynamin-mediated endocytosis, lipid rafts, and macropinocytosis. Species B viruses (RV-B3 and RV-B14) productively uncoat in endosomes of the lysosomal pathway. Transport of these to the compartment of uncoating is independent of microtubules; consequently, they uncoat in endosomal carrier vesicles (ECVs). In contrast, the species A viruses RV-A16 and RV-A89 require microtubules for transfer to the ERC where they productively release their RNA. Figure adapted from ref. [6].

Comparing the effect of low-molecular mass inhibitors specific for distinct steps in endocytosis on infection by representatives of species A and species B major receptor group RVs, we found that both were dependent on clathrin, dynamin, and cholesterol for cell entry (Figure 7). Dynasore, chlorpromazine, filipin, and blebbistatin all reduced, and NH_4_^+^ almost abolished, viral infection. The latter indicates that low pH is strongly promoting ICAM-1 ‘catalysed’ structural changes of the virion and RNA release [11]. Control experiments showed that cholesterol sequestering inhibited viral entry. Cytochalasin D and jasplakinolide inhibited RV-B3, RV-B14, RV-A16, and RV-A89, indicating their requirement for functional actin for uptake. However, whereas RV-B3, RV-B14, and RV-A16 did not infect when internalized at 20 °C, followed by neutralizing the endosomal pH and further incubation at 37 °C, RV-A89 replication occurred regardless of whether NH_4_^+^ was added prior to the temperature increase or not (Figure 4B). At first sight, this would suggest that a step after the formation of the A-particle, presumably RNA exit and/or its transfer through the endosomal membrane, is strongly temperature-dependent. However, when internalized at 20 °C, all viruses recovered from the cells were likewise infective, indicating that they had not transformed into subviral particles. Thus, the transformation, including RNA release, can only occur upon raising the temperature. Our current data show that the genomic RNA of RV-A89 does not arrive in the cytosol at 20 °C, but can do so upon returning to 37 °C, even at neutral endosomal pH.

What distinguishes RV-A89 from the other three RVs? The acid lability determined in vitro at 20 °C is similar for all four RVs (data not shown) and, as suggested by the experiment summarized in Figure 4C, the same appears to be the case in vivo. As already mentioned in our previous paper [14], RV-A89, as compared to RV-B14, is very strongly inhibited by recombinant soluble ICAM-1 (sICAM-1) with half maximal inhibitory concentration (IC_50_) of 70 nM and 3272 nM, respectively [37]. We, thus, hypothesized that these two serotypes might dissociate from ICAM-1 at different stations on their pathway through the endosomal system. This contention now becomes more likely by demonstrating that RV-A16 and RV-A89 are routed to the ERC; RV-A16 (IC_50_ = 81 nM) exhibits a similarly low IC_50_ value as RV-A89. In contrast, RV-B3 (IC_50_ = 4623 nM) and RV-B14 exhibit similarly high IC_50_ values; these viruses productively uncoat in endosomal compartments of the lysosomal route. As also discussed in our previous paper, the IC_50_ value does not distinguish between inhibition via competition for the receptor at the cell surface or via ‘catalysing’ the structural changes related to uncoating [37]. Nevertheless, RV-A16 cannot convert into its subviral particles at 20 °C, although it is routed to the ERC.

Is a secondary receptor involved? We have previously demonstrated that RV-A89 can be adapted to bind heparan sulphate, in addition to ICAM-1. This was brought about by repeated blind infection cycles alternating between ICAM-1-deficient Cos-7 cells and boosting in (ICAM-1-expressing) HeLa cells [17,38]. Such variants were finally able to use heparan sulphate as the sole receptor, but had become more acid-sensitive, presumably to compensate for the lack of the ‘catalytic’ activity of ICAM-1. Control experiments showed that the RV-A89 clone used in the present experiments could not grow in Cos-7 cells and, thus, behaved identically to the original wild-type strain. Currently, we cannot exclude with certainty that our RV-A89 uses, in addition to ICAM-1, heparan sulphate, or another cellular membrane protein as a co-receptor that is not able, on its own, to support binding and/or entry. Nevertheless, this is unlikely since the addition of excess heparan sulphate did not inhibit infection (data not shown). We also do not know whether the unique behaviour of RV-A89 is cell-type specific. To find out, additional experiments are necessary. Finally, the coordinated action of low pH and ICAM-1 on uncoating is not entirely clear. There might not only be a synergistic but also an anti-synergistic activity [11]. To find out it would be necessary to test ICAM-1-mediated inactivation of these four virus types at acidic pH. Apparently, only RV-A89 can be uncoated when first bound to ICAM-1 at 20 °C at mildly acidic pH and warming to 37 °C at neutral pH. Precedence for such ‘priming events’ exists in the case of influenza virus, whose nucleoprotein needs exposure to mildly low pH (6.5) followed by a more profound decrease of the pH to 6.0 and a switch from Na^+^ to K^+^ in maturing endosomes [39].

Our data clearly show that extrapolation and generalization of experimental results obtained with one particular virus onto closely-related other ones is only partly legitimate and must be done with extreme care. Further research will show how many pathways into a host cell might be exploited by other members of the genus *Enterovirus* and of various representatives of even the same species, A, B, and C, of human rhinoviruses.
